# Volunteering in medical school during the pandemic: a solution for teaching

**DOI:** 10.15694/mep.2020.000114.1

**Published:** 2020-05-28

**Authors:** Daniela Mendes Chiloff, Victor Muniz de Freitas, Natália Borges Cardin, Camila Bianchi Matiuzzi, João Aléssio Juliano Perfeito, Emilia Inoue Sato, Marcus Vinicius Malheiros Luzo, Manoel João Batista Castello Girão, Aécio Flávio Teixeira Góis

**Affiliations:** 1 Escola Paulista de Medicina - UNIFESP

**Keywords:** Medical education, coronavirus, pandemic, volunteering, medical school

## Abstract

This article was migrated. The article was marked as recommended.

**Introduction:** On March 18, 2020, in face of COVID-19 pandemic and the suspension of in-person activities by the Board of the Paulista School of Medicine (EPM), students on clinical rotations (5th and 6th grades) organized themselves to support the local community and the Hospital São Paulo complex.

**Method:** The construction of the Volunteering-EPM was, despite fast, progressive, following as required by the Hospital São Paulo-Escola Paulista de Medicina complex.

**Results:** After one week, Volunteering-EPM added more than 100 students and the unconditional support of professors. The quantifiable results enable an adequate supply of resources to the hospital complex. However, the biggest impact was the moment of solidarity promoted by the initiative.

**Discussion:** Volunteering enabled unique experiences for those involved, enhancing students and professor’s skill sets otherwise not developed in medical school. Emphasizing the humanitarian view of medicine improved employee and community health access and welfare.

**Conclusion:** The speed with which actions were implemented and their impact on the community shows the ability for transformation of the volunteers. The immediate demands have been solved. In medium and long term, the project continues to respond to the new demands of the hospital.

## Introduction

On March 18, 2020, with the progression of the new coronavirus in Brazil and the city of São Paulo being the epicentre of this pandemic in the country ( *Coronavírus Brasil*, no date), the Board of the Paulista School of Medicine of the Federal University of São Paulo (EPM-UNIFESP), in accordance with the dean of the medical course, suspended in-person activities of the medical school. Immediately, 5th and 6th grade students turned to teachers to express their desire to actively and voluntarily assist in fighting the disease and supporting the Hospital São Paulo-Escola Paulista de Medicina (HSP-EPM) complex ( Whelan *et al.*, 2020).

Few days after, a structure composed of students and professors was organized, in addition to the EPM board. It was the prelude to Volunteering-EPM. Synchronically, other similar initiatives have been created and encouraged in other universities around the world, confirming the importance of voluntary activities for medical education at this time ( Soled *et al.*, 2020).

An action plan was drawn to foresee the problems that the hospital complex would face and their possible solutions. As the interns are part of the operation of the hospital, the suspension of in-person activities imposed a lack of human resources for the health care and consequent overload of those who would continue on the front line. Voluntary action by students was necessary, even though not working directly at the *front.*

In addition to the overload of human resources, the sudden increase in demand and the new needs for Personal Protective Equipment (PPE) cautioned the risk of professionals’ exposure due to a shortage of PPE. The collection of resources, either financial or material, was listed as the initial priority of the new organization.

With the consolidation of Volunteering-EPM as a support entity for the HSP-EPM complex, the following objectives were determined: (1) protection and support for professionals in the HSP- EPM / UNIFESP complex; (2) monitoring, guidance and awareness of the local community; (3) support to the physical and mental health of EPM-UNIFESP students and residents.

## Methods

Volunteering-EPM was structured according to the demands of the HSP-EPM complex. On March 18, 2020, four coordinators were defined among the students, integrated with a group of professors. A schedule of planning meetings was established, which were held in a large amphitheatre, following the recommendations for distance and use of masks.

In view of the epidemiological situation, students could be asymptomatic carriers of COVID-19 and represent a further expose to patients. Direct contact with patients, therefore, should be restricted ( Rose, 2020). Thus, distance activities and face-to-face activities were established, also not including direct contact with patients. The first actions designed focused on the collection of PPEs, the mental health of students and residents, and scenarios and initiatives in which students could contact patients indirectly.

The search for materials and PPE for front-line professionals required great creativity from all the volunteers involved. A fundraising campaign was launched in conjunction with the EPM Board, both in terms of money and materials. Volunteer students were responsible for organizing and receiving donated materials. A project was also started to develop alternatives to commercial products, such as face shields, aprons and disposable masks.

The need to avoid the unnecessary flow of people in the hospital and the importance of monitoring patients suspected of COVID-19 on more than one occasion, originated the project of telephone follow-up of patients treated at the Respiratory Infection Unit (UIR), set up exclusively for initial care of suspected cases of COVID-19. The practice of telemonitoring ( Ribeiro, 2020) does not expose interns to traditional risks and allows them the chance to practice under the supervision of a teacher. In addition, it allows experienced doctors to occupy the front line, while the rear is composed by students still in training.

In a short time, the population’s involvement and support to the project made new actions also essential. The local community needed to be included. Underprivileged communities in the surrounding area needed guidance on the health situation, assistance in obtaining hygiene and food items and, above all, a health structure that would welcome them. These new demands expanded the objectives initially proposed and triggered a series of actions for vulnerable populations, such as guidance and assistance projects regarding influenza vaccination for the elderly and vulnerable population( *Campanha de Vacinação contra a Influenza 2020 | Secretaria Municipal da Saúde | Prefeitura da Cidade de São Paulo*, 2020). The HSP-EPM complex needed to assume its social and preventive role within the local community and, therefore, reduce the future demand for health services.

With the impact produced and with a dynamic of preparation for a real combat action, new demands arose, establishing the Volunteering-EPM as an active and integral part of the university and hospital team.

## Results

With about a month in progress, the volunteer program has 110 students of the internship, already expanding to students from the 1st to the 4th grades and adds several positive results on its various initiatives. With 14 projects executed, the reach of the actions ranges from thousands of doses of vaccine applied to the amount collected in donations, in cash or in materials ( Table 1). In addition to what has already been achieved, the project’s main outcome is the moment of solidarity it has started, involving more and more participants and impacting far beyond the hospital walls.

**Table 1.** Volunteering-EPM activities in progress

**Project**	**Goals**	**Actions**	**Number of Volunteers**
**Monitoring Respiratory Infections Unit of Hospital São Paulo (UIR-HSP-COVID-19)**	Monitor the evolution of the patients attended, promoting longitudinal care, solving doubts and avoiding unnecessary returns to the Emergency Room, in addition to evaluating patients who need to return.	Calls to patients previously seen at the UIR; providing guidance on care and warning signs; referral to the UIR, if necessary.	34
**Collection of donations**	Receive and organize material donations.	Receipt, cataloguing and organization of donations in the materials warehouse.	51
**Aid for students in situations of socio-economic vulnerability**	Assist students from Escola Paulista de Medicina and Escola Paulista de Enfermagem who are in a situation of socio-economic vulnerability.	Virtual registration of students in situations of socio-economic vulnerability; provision of basic food items for those registered.	3
**Securing PPE producers**	Active search for companies or people who could produce PPE.	Active search for companies, micro entrepreneurs, students and freelancers who could produce PPE’s according to the regulations of the Ministry of Health.	15
**Food collection campaign**	Collection of food for the Mário Cardim Community (vulnerable local community).	Allocation of food collection posts in condominiums in the region and donation of food collected to the Mário Cardim Community.	5
**Influenza vaccination campaign 2020**	Assist the HSP nursing team in the 2020 Influenza Vaccination Campaign.	Assist the nursing team, organizing lines and applying vaccines, in vaccination campaigns carried out at HSP, in vulnerable communities and in Military Police Battalions.	40
**Mapping of COVID-19 suspects among students**	Identification of students who have symptoms compatible with the new coronavirus to provide appropriate medical or non-medical support.	Elaboration and feasibility of filling out the virtual register for students with flu-like symptoms and providing adequate support.	2
**Community educational material**	Community awareness.	Production of flyers and visual arts that are easy to understand, with the aim of raising awareness and guiding the community about coronavirus care.	12
**Educational material for healthcare professionals**	Produce institutional videos and make material accessible to students, doctors and patients from all over Brazil.	Production of instructional videos, together with EPM teachers.	12
**Social media**	Disclosure of Volunteering-EPM.	Creation of own website and Instagram with daily update.	8
**Scientific work: Blood plasma**	To evaluate the therapeutic effect of the use of convalescent plasma in critically ill patients admitted by COVID-19.	Telephone contact with patients with positive PCR test for coronavirus (SARS-CoV-2), aiming at the donation and collection of plasma.	11
**Scientific work: Hydroxychloroquine** **Project Mário Pinotti II**	To evaluate the effects of Chloroquine / Hydroxychloroquine on COVID-19 infection in patients with immune-mediated rheumatic diseases.	Serial phone calls with patients who follow up at Rheumatology services throughout Brazil and data collection on symptoms or contacts with suspected or confirmed cases of COVID-19.	26
**Valuing employees**	Promote a welcoming work environment for our employees, both in the front line and in the administrative part.	Posts of gratitude and appreciation towards employees and active search for donations of meals to employees during their periods of activity.	7
**Cultural Project**	Promote physical and mental health of the EPM-UNIFESP community.	Cultural contest with the theme “COVID-19 pandemic: the quarantine for the individual”.	10

The various donation campaigns originated within the volunteer by its diverse members (students, professors and EPM board) managed to raise more than two million and five hundred thousand reais and a large amount of materials. All of these financial resources were used to purchase equipment and supplies to assist the population, either during the pandemic or as a positive legacy, improving the infrastructure that will continue to serve the population of the state of São Paulo. In addition, sewing ateliers and 3D printing networks were jointly adapted to produce PPE and supply health facilities.

The telemonitoring project created to monitor suspected COVID-19 patients treated by UIR, made more than 1600 phone calls in one month. It reached and followed most of the patients treated by the UIR, providing safe information and guidance, sheltering and calming those who were anxious and identifying those at greatest risk who required a new evaluation. What was a voluntary project became, in a short period, a formal and integral part of the monitoring of this unit.

The participation of volunteer students facilitated the organization and application of more than 12,000 doses of the Influenza Vaccine 2020, with specific actions aimed for the elderly population, the vulnerable local community and several battalions of the Military Police, in addition to the community of healthcare employees.

Beyond the numerical effects, volunteering opened the eyes to new possibilities for collaboration. Withdrawn teachers, both symptomatic and at risk, started to think about projects, linked to their departments, that, in some way, could support the community and HSP professionals.

The various actions focused on employees, in addition to guaranteeing PPE’s, promoted an improvement in mental health through the care they inspired. Donated dinners and chocolates for Easter were much more than food, but demonstrations of affection with those working on the front lines.

## Discussion

The feeling of helplessness in face of the pandemic was replaced by an impetus and a new purpose. Occupying part of their days with actions that positively impact society, volunteer students improved their mental health during the period of social distance, as also reported by other similar programs ( Soled *et al.*, 2020).

For members of the coordination of Volunteering-EPM, the opportunity to build an extracurricular organization from the beginning and to mediate the relationship between students, professors and hospital dynamics was unique. The experience brings several technical teachings, such as people management, educational production, video editing and creation of illustrations. The biggest gain, however, was to bring out the humanitarian view of medicine, sometimes so overlooked in formal medical education.

The experiences of being part of something bigger and with the objective of alleviating the needs and suffering of others, even if in a small degree, are great opportunities in graduation. The students involved in the project experienced the gratitude of patients, professors and staff.

For professors who have followed the project since the beginning, Volunteering-EPM has become an example and source of enthusiasm. “Dear students, fantastic things happened this month and a lot did we learn together in these few days. You are setting an example and encouraging us all.” Messages such as this one, from a professor of the organization, summarize the impact of the actions.

For doctors and hospital staff, Volunteering-EPM meant support and refuge, expressed by the head of the Pulmonology discipline: “At many times, when I arrived at the meeting, I wanted a hug. I couldn’t, two meters away. But I felt the embrace in the eyes, the words and the actions.”

The speed with which the actions took place inspired the emergence of other similar actions in other centres, through the exchange of experiences between students and professors from different universities. The mobilizing power of actions like this led to the creation of similar ones, amplifying the transforming power of people interested and committed to making difficult situations a little better.

The greatest learning, in the end, was summed up by a donor who sent cakes to the on-duty staff during the Easter period. The delivery was accompanied by the message: “I don’t know how to sew masks, but today I did my best with a lot of love”. Each volunteer and employee have a way to help and any action is welcomed. The solidarity network built in this period will be a differential to overcome this pandemic.

## Conclusion

The results obtained so early with the initiative demonstrated the capacity for transformation that groups of interested and committed people are able to obtain, especially in a context of sudden disorganization and helplessness. Volunteering became a valid solution for both the immediate demands, such as lack of PPE, and for the long-term medical education at the Paulista School of Medicine.

## Take Home Messages

• Volunteering is a teaching alternative during the pandemic.
• Volunteer students responding to the needs of the hospital and community.
• Networking and team-based initiatives are effective ways of engaging students.
• Empowering students’ leadership during its schooling improves educational results.
• Solidarity is a value to be rescued at the humanitarian medical education.

## Notes On Contributors

**Daniela Mendes Chiloff**: Fifth year medical student of Paulista School of Medicine of the Federal University of São Paulo; ORCID ID: https://orcid.org/0000-0002-6003-2106.

**Victor Muniz de Freitas**: Fifth year medical student of Paulista School of Medicine of the Federal University of São Paulo; ORCID ID: https://orcid.org/0000-0002-3842-818X.

**Natália Borges Cardin**: Sixth year medical student of Paulista School of Medicine of the Federal University of São Paulo.

**Camila Bianchi Matiuzzi**: Sixth year medical student of Paulista School of Medicine of the Federal University of São Paulo.

**João Aléssio Juliano Perfeito**: Associate Professor of the Department of Surgery at Paulista School of Medicine of the Federal University of São Paulo; ORCID ID: https://orcid.org/0000-0001-5958-2541.

**Emilia Inoue Sato**: Full Professor of Rheumatology, chief of the Department of Medicine at Paulista School of Medicine of the Federal University of São Paulo, Master in Rheumatology by the Pan American League of Association of Rheumatology; ORCID ID: https://orcid.org/0000-0001-8540-014X.

**Marcus Vinicius Malheiros Luzo**: Adjunct Professor of the Department of Orthopaedics and Traumatology and Coordinator of the Department of Orthopaedics at Paulista School of Medicine of the Federal University of São Paulo; ORCID ID: https://orcid.org/0000-0003-1345-6915.

**Manoel João Batista Castello Girão**: Full Professor of Department of Gynaecology at Paulista School of Medicine of the Federal University of São Paulo and Director of the Paulista School of Medicine of the Federal University of São Paulo; ORCID ID: https://orcid.org/0000-0002-3379-5802.

**Aécio Flávio Teixeira Góis**: Adjunct Professor of Discipline of Emergency Medicine at Paulista School of Medicine of the Federal University of São Paulo, coordinator of the medical course at Paulista School of Medicine. ORCID ID: https://orcid.org/0000-0003-0217-1463.
