# Supplementary material for: Volunteering in medical school during the pandemic: a solution for teaching
Source: MedEdPublish (2016). 2020 May 28;9:114. [Version 1] doi: 10.15694/mep.2020.000114.1 (PMC10702680; doi:10.15694/mep.2020.000114.1)
Supplement: Peer review history

# Reviewer report: Reviewer response for version 1

**DOI:** 10.21956/mep.20089.r30937

**Published:** 2020-10-17

**Authors:** Ken Masters

**Affiliations:** 1 Sultan Qaboos University

This review has been migrated. The reviewer awarded 4 stars out of 5 An interesting and useful paper dealing with medical students’ volunteering for a wide range of healthcare-related tasks during the Corona pandemic. The authors have clearly demonstrated that, with the required precautions, medical students can assist a health system when it becomes burdened with such an event.The paper does, however, suffer from a number of language errors and expressions that can make reading difficult. At times, the meaning is very difficult to determine. I would recommend that future papers be carefully proof-read to reduce errors.For a future, follow-up paper, it would be useful to see the long-term effects, on the system and on the students. Initial volunteerism is usually very enthusiastic, but there is the risk of burnout as students become deeply immersed in the process, and either their other work begins to suffer, or they try to do too much at once. So, a follow-up paper, seven or eight months into the pandemic would also be interesting, and would hold valuable lessons for anyone who wished to replicate this work.

Reviewer Expertise:

NA

No decision status is available

# Reviewer report: Reviewer response for version 1

**DOI:** 10.21956/mep.20089.r30938

**Published:** 2020-06-01

**Authors:** P Ravi Shankar

**Affiliations:** 1 American International Medical University

This review has been migrated. The reviewer awarded 4 stars out of 5 This is an interesting manuscript about volunteering at a Brazilian medical school. The authors have described the organization of the volunteer force and its formation and motivation very well. Table 1 describes the different volunteering activities at the medical school and in the community. The wide range and reach of these activities are well described. The benefits of volunteering have been well described. I am happy to note that most of the authors are medical students. There are few issues with the use of language which can be resolved. This article will be of interest to medical educators in a variety of settings.

Reviewer Expertise:

NA

No decision status is available
